# Supplementary material for: Artificial intelligence’s contribution to biomedical literature search: revolutionizing or complicating?
Source: PLOS Digit Health. 2025 May 12;4(5):e0000849. doi: 10.1371/journal.pdig.0000849 (PMC12068611; doi:10.1371/journal.pdig.0000849)
Supplement: Attachment — Submitted filename: Response Letter.docx [file pdig.0000849.s003.docx]

Editor comments:

***1****. We have amended your Competing Interest statement to comply with journal style. We kindly ask that you double check the statement and let us know if anything is incorrect.*

Thank you for amending the Competing Interest statement. We have reviewed the revised statement and can confirm that it is correct.

***2.*** *Please provide an Author Summary. This should appear in your manuscript between the Abstract (if applicable) and the Introduction, and should be 150–200 words long. The aim should be to make your findings accessible to a wide audience that includes both scientists and non-scientists. Sample summaries can be found on our website under Submission Guidelines.*

We appreciate your feedback and would like to clarify that our original manuscript includes an Author Summary, located after the Abstract on page 3, lines 32–47. Please let us know if any adjustments are needed to better align with the journal's expectations.

***3****. In the online submission form, you indicated that “Any additional information required to reanalyze the data reported in this paper is available from the lead contact upon request.”*

*All PLOS journals now require all data underlying the findings described in their manuscript to be freely available to other researchers, either*

*1. In a public repository,*

*2. Within the manuscript itself, or*

*3. Uploaded as supplementary information.*

*This policy applies to all data except where public deposition would breach compliance with the protocol approved by your research ethics board. If your data cannot be made publicly available for ethical or legal reasons (e.g., public availability would compromise patient privacy), please explain your reasons by return email and your exemption request will be escalated to the editor for approval. Your exemption request will be handled independently and will not hold up the peer review process, but will need to be resolved should your manuscript be accepted for publication. One of the Editorial team will then be in touch if there are any issues.*

We are fully committed to complying with *PLOS Digital Health*'s data availability policy. To promote transparency, we have provided a supplementary document containing all ChatGPT conversation histories collected during our study.

***4****. Some material included in your submission may be copyrighted. According to PLOS’s copyright policy, authors who use figures or other material (e.g., graphics, clipart, maps) from another author or copyright holder must demonstrate or obtain permission to publish this material under the Creative Commons Attribution 4.0 International (CC BY 4.0) License used by PLOS journals. Please closely review the details of PLOS’s copyright requirements here: PLOS Licenses and Copyright. If you need to request permissions from a copyright holder, you may use PLOS's Copyright Content Permission form.*

*Please respond directly to this email or email the journal office and provide any known details concerning your material's license terms and permissions required for reuse, even if you have not yet obtained copyright permissions or are unsure of your material's copyright compatibility.*

*Potential Copyright Issues:*

*Figure 2: Please confirm whether you drew the images / clip-art within the figure panels by hand. If you did not draw the images, please provide (a) a link to the source of the images or icons and their license / terms of use; or (b) written permission from the copyright holder to publish the images or icons under our CC-BY 4.0 license. Alternatively, you may replace the images with open source alternatives.*

Thank you for addressing potential copyright issues. We have carefully reviewed our figures and replaced any copyrighted materials in Figure 2 with hand-drawn graphics created by ourselves. This ensures full compliance with *PLOS Digital Health*'s copyright policy.

***5.***  *I suggest improving the pubmed search strategy, clarifying the the use of a prompt as a search strategy with an acronym instead of a keyword search.*

Thank you for this thoughtful suggestion. Acronyms such as PICO, SPIDER, and SPICE are well-established structured search strategies that can refine PubMed searches and improve literature retrieval. Our study aimed to compare search outcomes across different search engines and ChatGPT from the perspective of an average end-user in everyday research scenarios. While structured search strategies can enhance PubMed searches, they are not commonly utilized in routine biomedical literature searches by general users. However, we truly appreciate this valuable point and have highlighted the role of structured search strategies in our discussion section to provide a more comprehensive perspective.

Specifically, to address the reviewer’s comment regarding our search strategy, we have expanded on this in lines 100-104: “It is notable that PubMed offers functions for optimizing search results, such as the use of Boolean operators or filter results by journal categories and study types (Fig 1C). For this study, we did not employ optimization strategies, as our goal was to compare search outcomes from the perspective of an average end-user in typical research scenarios.”

***6.***  *I suggest improving the quality of the figures, as the details are difficult to read in their current form. Clearer visuals would significantly aid reviewers and readers’ comprehension.*

Thank you for the helpful feedback. We recognize the importance of clear and high-quality figures to aid comprehension, and we appreciate the editor’s keen eye in pointing this out. To address this concern, we have made substantial improvements to the quality and readability of our figures. Since the finer details in the ChatGPT/other search engine responses were not meant to be the primary focus, we have removed unnecessary information, blurred out less relevant text, and incorporated chat bubbles to magnify and highlight key information. Below are the specific modifications we have made to enhance each figure:

Figure 1:

1. Removed extraneous details from the original search result screenshots (Google, PubMed, Google Scholar, and ChatGPT) to improve clarity.
2. Blurred out the full search results to minimize distractions while retaining the context of the search process.
3. Added pie charts to visually depict quantitative differences.
4. Incorporated chat bubbles to highlight and magnify the most relevant information for readers.

Figure 2:

1. Removed non-essential details from the ChatGPT responses to improve clarity.
2. Used chat bubbles to highlight key information, including the exact prompt used in the prompt engineering example and correct reference links
3. Replaced the drawings with hand-drawn graphics to avoid copy-right issues.

Figure 3:

1. Increased the text size of the exact prompt used in the prompt engineering example to enhance readability.

Figure 4:

1. Blurred out non-essential details in the ChatGPT response screenshot to reduce visual clutter.
2. Replaced them with speech bubbles and extended text boxes to emphasize key messages conveyed by the figure.

***7.*** *The Methods section would benefit from reorganization, as some methodological descriptions appear within the Results and Discussion sections.*

Thank you for your feedback. We have reorganized the Methods section and revised the manuscript accordingly. Some methodological details, such as how results obtained from search engines (Google, Google Scholar, PubMed) were evaluated, have been moved to the Methods section for clarity. We have retained certain methodological descriptions in the Results and Discussion sections where they are essential for clarification and comparison (e.g., iteration numbers differing for each query). Additionally, we have removed some repeated details to improve readability and conciseness. Please let us know if further adjustments are needed. We appreciate your guidance in strengthening the manuscript.

Methods Section

Line 283-291: “Each query was tested in 5 to 10 independent iterations, where each iteration was conducted as a new ChatGPT session to prevent context retention bias. To assess for accuracy, we manually verified whether the manuscript titles in ChatGPT responses match actual publications that can be identified in Google/Google Scholar/PubMed repository. For cases where a hyperlink is provided in the response (e.g., from web-browsing GPT, plugins or custom GPT), we added an additional criterion to check if the provided link correctly directed to the article cited in its response. Note that an “accurate reference” in the scientific context also includes additional criteria such as correct authorship, publication date, and journal name. We did not include these in our criteria as we focused on assessing ChatGPT’s utility in real-world scenarios for identifying literatures..”

Line 292-296: “A response can only be used to assess for relevancy if it provided accurate reference as defined above. We proofread each individual article that was returned and checked if it contained relevant information pertaining to the query in testing. Responses from all the iterations for a specific query were compared to evaluate consistencies across independent sessions. For queries conducted on search engines (i.e Google, PubMed, and Google Scholar), we proofread and evaluated the top ten results.”

Reviewer #1 comments:

***1.*** *Has the statistical analysis been performed appropriately and rigorously?*

*Reviewer #1: No*

We sincerely appreciate the reviewer’s feedback on our analytical approach. We acknowledge that while we included some quantitative evidence to assess consistency, accuracy, and relevance across multiple iterations, our methodology was primarily descriptive rather than based on formal statistical testing. This was a deliberate choice, as our goal was to provide a practical evaluation of AI-assisted literature searches rather than conduct a statistically inferential study. We hope this clarifies our approach, but we are open to additional suggestions if the reviewer feels a specific statistical method could enhance the manuscript.

***2.*** *While the comparison with PubMed and Google is insightful, the manuscript states that no search optimization strategies (e.g., Boolean operators) were used in PubMed to ensure fairness. However, PubMed’s strength lies in its ability to narrow results using advanced search functionalities, not using these functions could understate the potential of conventional methods, mainly when additional settings such as prompt engineering or web search are included in ChatGPT.*

Thank you for this insightful comment. As this concern aligns with editor comment #5, we have addressed it in detail in that section. Briefly, while structured search strategies such as PICO, SPIDER, and SPICE can refine PubMed searches, they are not commonly used by general users in routine biomedical literature searches. Our study focused on comparing search outcomes from an average end-user perspective. However, we have now expanded our discussion to acknowledge the role of structured search strategies and their potential impact. Please refer to our response in editor comment #5 for further details.

***3.*** *While plugins and prompt engineering are tested, the explanation of how these modifications improved or failed is somewhat superficial. For example, how exactly does “prompt engineering” improve accuracy and relevance? A more granular analysis of the specific improvements introduced by each augmentation would strengthen the conclusions. Additionally, sharing the prompts used could improve the reliability and reproducibility of the experiments.*

Thank you for this valuable feedback. We have revised the manuscript to include deeper explanations and more granular analysis to explain how augmentation strategies such as the use of web-browsing functionality, plugins, prompt engineering, and custom GPTs can introduce improvements. We have also added additional references in our manuscript to help direct readers to academic literature on large language models that would further deepen their understanding. Below are the specific modifications we made to the manuscript:

Results and Discussions

Line 144-146: “The incorporation of web-browsing functions enabled the LLM to access more current information available on the internet and improved search responses.”

Line 152-158: “These prompt refinements align with the prompt engineering strategy of providing clear instructions, as recommended by OpenAI. A clearer and more detailed prompt improves the LLM’s response by narrowing the scope and reducing errors caused by ambiguity. Additionally, the extra information acts as a filter, guiding the LLM to exclude studies that do not meet the additional criteria. However, it is important to note that challenges with inconsistency and accuracy continued.”

Line 169-171: “This improvement stems from Scholarly’s ability to access specialized academic repositories and its built-in constraints, ensuring the LLM to only retrieve data exclusively from these sources.”

Line 177-180: “This improvement may be due to Consensus’s functionality of accessing information specifically from the Semantic Scholar database, using a fine-tuned LLM specifically trained to understand and summarize research articles, and an advanced in-built search algorithm that improves literature search responses.”

Line 220-224: “Recently, it was suggested that ChatGPT may generate better-quality responses when it is prompted to reason through steps by splitting complex tasks into simpler, intermediate tasks. This prompting method, termed “Chain-of-Thought Prompting” can enable the LLM to focus on fewer variables at a time with more computation, resulting in lower error rates, and improving the LLM’s reasoning ability.”

We recognize that though some of the prompts we used were included in the text, others in the figures were unclear due to resolution issues. To resolve this, we have significantly improved the quality of our figures to explicitly highlight the exact prompts we have used for each search scenario. This was done by magnifying and emphasizing the prompt texts through enlarging the fonts and the use of speech bubbles for an enhanced resolution in all our figures.

***4.***  *Although the manuscript mentions "consistency," "accuracy," and "relevance" as evaluation metrics, these terms are not defined with clear, measurable criteria. Providing concrete definitions or scoring systems for these terms would allow for more rigorous assessment. For instance, how is "accuracy" quantified when ChatGPT provides a reference—what makes a reference “accurate” beyond its correct citation formatting?*

Thank you for your comment. We agree that  "consistency," "accuracy," and "relevance" evaluation terms were not defined with clear measurable criteria. We have edited the manuscript to include two additional paragraphs in the methods section to provide concrete definitions of our evaluation metrics.

For the “accuracy” metric, we considered a response to be accurate if the manuscript title provided by the ChatGPT response matched an actual publication that could be identified in Google/Google Scholar/PubMed repository and if the provided hyperlink correctly directed to the article cited. We acknowledge that in the scientific context, an accurate reference also includes correct authorship, publication date, and journal name. However, these elements were not included in our criteria, as our goal and focus were to assess ChatGPT’s utility in real-world literature searches. If ChatGPT can correctly identify and direct users to the relevant literature, researchers can then verify the complete reference details at the original source.

Below are the additional details we have incorporated into the methods section:
Line 270- 283: “Each query was tested in 5 to 10 independent iterations, where each iteration was conducted as a new ChatGPT session to prevent context retention bias. To assess for accuracy, we manually verified whether the manuscript titles in ChatGPT responses match actual publications that can be identified in Google/Google Scholar/PubMed repository. For cases where a hyperlink is provided in the response (e.g., from web-browsing GPT, plugins or custom GPT), we added an additional criterion to check if the provided link correctly directed to the article cited in its response. Note that an “accurate reference” in the scientific context also includes additional criteria such as correct authorship, publication date, and journal name. We did not include these in our criteria as we focused on assessing ChatGPT’s utility in real-world scenarios for identifying literature.

A response can only be used to assess for relevancy if it provided accurate reference as defined above. We proofread each individual article that was returned and checked if it contained relevant information pertaining to the query in testing. Responses from all the iterations for a specific query were compared to evaluate consistencies across independent sessions. For queries conducted on search engines (i.e Google, PubMed, and Google Scholar), we proofread and evaluated the top ten results.”

***5.*** *The manuscript limits each search scenario to five or ten iterations, which is a relatively small sample size for assessing the variability in outputs. This is especially important when evaluating AI, as its performance can vary significantly depending on input nuances. Expanding the number of iterations per scenario and including other statistical measures could provide a more robust understanding of ChatGPT's limitations and strengths.*

Thank you for this insightful comment. We agree that increasing the number of iterations could provide a more comprehensive assessment of variability in ChatGPT’s responses. However, due to the rapid evolution of AI models, some of the versions we tested are no longer available, making it impossible to re-run the exact same experiments for expanded sampling. For instance, certain ChatGPT models and plugins used in our study have since been updated, replaced, or removed from public access. As a result, while we recognize the value of additional iterations, replicating our original testing conditions is no longer feasible.

To address this concern, we have explicitly acknowledged this limitation in the discussion section and emphasized that our findings provide a snapshot of ChatGPT’s performance at the time of testing. Additionally, we have suggested that future studies consider real-time benchmarking of AI search tools to account for model updates and performance variability over time.

Line 255- 262: “This manuscript provides a snapshot view of LLM’s utility in literature searching at the time of testing in early 2024. As of March 2025, LLM and generative AI’s utility in literature search is no longer solely available in the form of conversational AI such as ChatGPT. Many of the search engines referred to “conventional” in this manuscript such as Google have already incorporated AI features to enhance literature searches available to users such as AI-generated summaries in Google responses. This manuscript evaluated ChatGPT’s utility by conducting search scenarios in 5 – 10 independent iterations. As LLMs quickly advance, we suggest future studies to increase the number of iterations for a more holistic understanding of the limitations of ChatGPT across different search scenarios.”

***6.*** *The quality of the images in the manuscript needs improvement, as they are difficult to review in their current form. Additionally, the accompanying descriptions lack clarity and should provide more detailed explanations to ensure the figures effectively support the findings and are easily interpretable by readers.*

Thank you for this insightful comment. As this concern aligns with editor comment #6, we have addressed it in detail in that section. Specifically, we have made substantial improvements to the clarity and readability of our figures by removing unnecessary details, enhancing key information with chat bubbles, increasing text size, and adjusting formatting for better visibility. These modifications ensure that key elements are more easily interpretable for readers. Please refer to our response in editor comment #6 for a more comprehensive discussion of this point.

Review #2 Comments:

***1.*** *Prompt engineering enhances quality and relevance of the responses, but it needs multiple iterations. In addition to the persistence of inconsistency and inaccuracy mentioned in the paper, it can mean the infusion of bias into the research structure and this bias might double fold with the increased iterations. The continuously changing research trends in healthcare also prompt continuous update of the engineered prompts in the deployment of ChatGPT.3*

Thank you for this insightful comment. We recognize the concern that repeated iterations of prompt engineering may introduce bias into the research structure, particularly if conducted within the same ChatGPT session. However, in our study, each iteration was performed as a new and independent ChatGPT session, meaning that previous prompts and responses did not influence subsequent queries. This approach was intentionally designed to prevent context retention bias, ensuring that each response was generated without any prior influence.

To clarify this further, we have explicitly emphasized in our methods section that all iterations were conducted in separate chat sessions to mitigate potential biases introduced by ChatGPT’s memory within a conversation.

Methods Section

Line 283-284: “Each query was tested in 5 to 10 independent iterations, where each iteration was conducted as a new ChatGPT session to prevent context retention bias.”

***2.*** *ChatGPT is already being tried for integration with the conventional search engines. 4 Conventional search engines including Google, Microsoft Bing and others are already implementing functionalities like ChatGPT to make their search more conversational. So, conventional search engines mentioned in the study are no longer conventional.*

Thank you for this excellent observation. At the time we conducted our analyses, AI-assisted search functionalities had not yet been widely incorporated into conventional search engines. However, we recognize that search engines such as Google and Microsoft Bing have since begun integrating conversational AI capabilities, fundamentally changing the landscape of online literature searching. This is a valuable point, and we have highlighted it in our discussion section to acknowledge how AI-driven search features are evolving and how this may impact future comparisons between ChatGPT and conventional search engines.

Line 250-253: “As of March 2025, LLM and generative AI’s utility in literature search is no longer solely available in the form of conversational AI such as ChatGPT. Many of the search engines referred to “conventional” in this manuscript such as Google has already incorporated AI features to enhance literature searches available to users such as AI-generated summaries in Google responses.”
